# Supplementary material for: Design, Synthesis and Evaluation of Novel Molecular Hybrids between Antiglaucoma Drugs and H2S Donors
Source: Int J Mol Sci. 2022 Nov 9;23(22):13804. doi: 10.3390/ijms232213804 (PMC9695456; doi:10.3390/ijms232213804)
Supplement: Supplementary file 1 [file ijms-23-13804-s001.zip › ijms-1980558-supplementary.pdf]

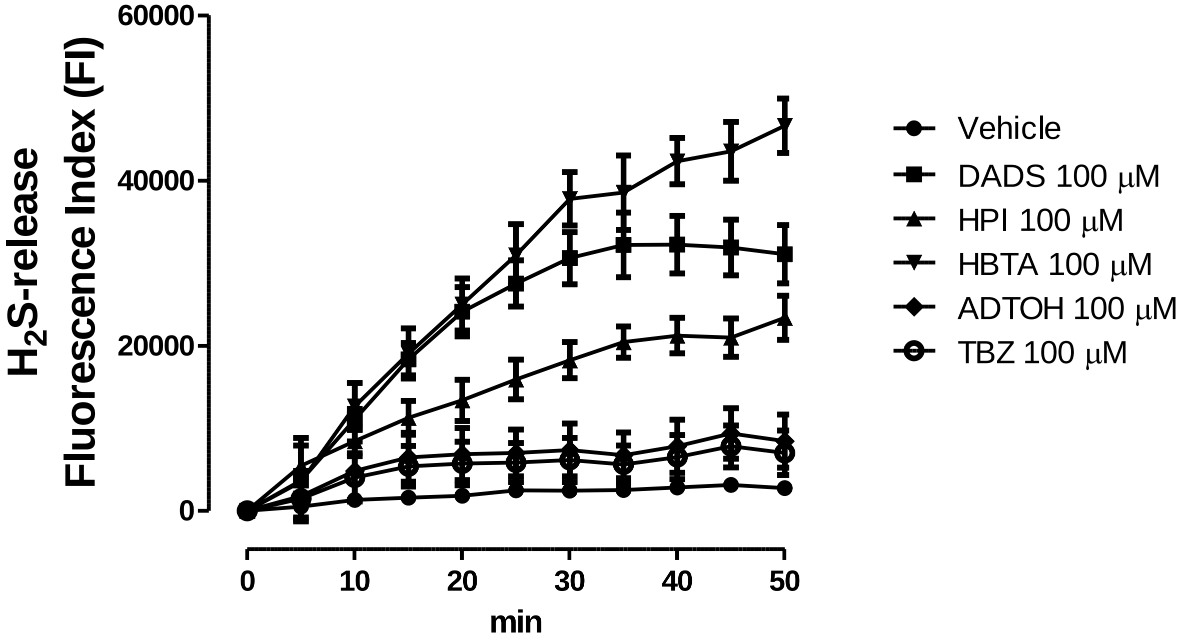

Figure S1. Fluorometric recording of H_2S released by vehicle, DADS 100µM, HPI 100µM, HBTA 100µM, ADTOH 100µM and TBZ 100µM for 50 minutes; the increase of H_2S is expressed as fluorescence index (FI).

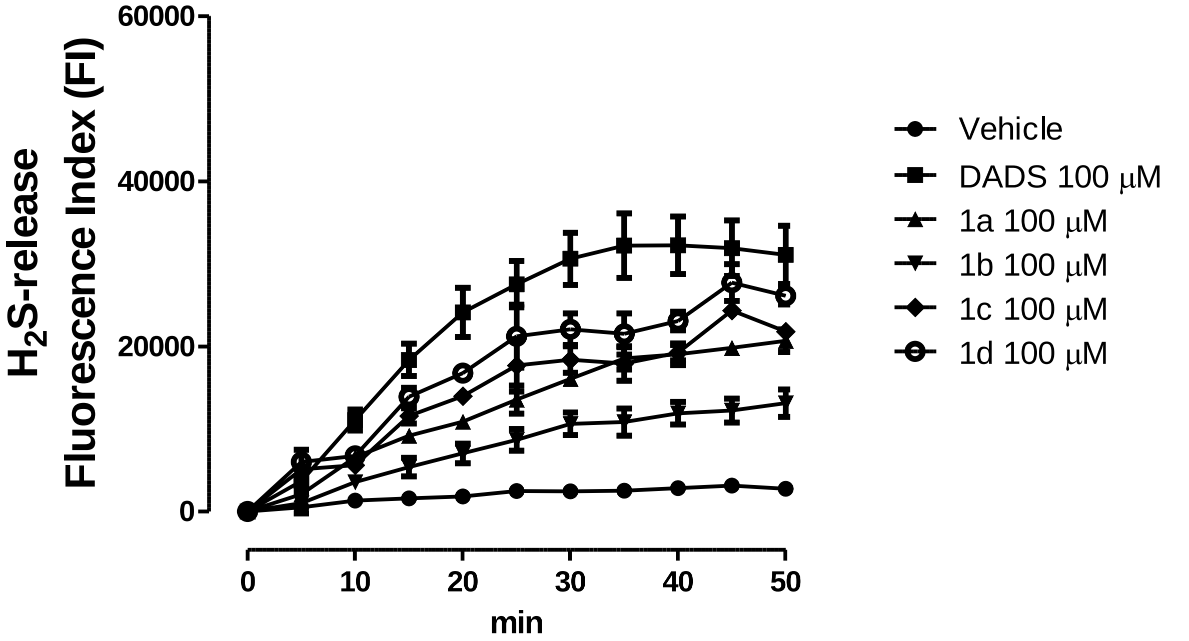

Figure S2. Fluorometric recording of H_2S released by vehicle, DADS 100µM, 1a 100µM, 1b 100µM, 1c 100µM and 1d 100µM for 50 minutes; the increase of H_2S is expressed as fluorescence index (FI).

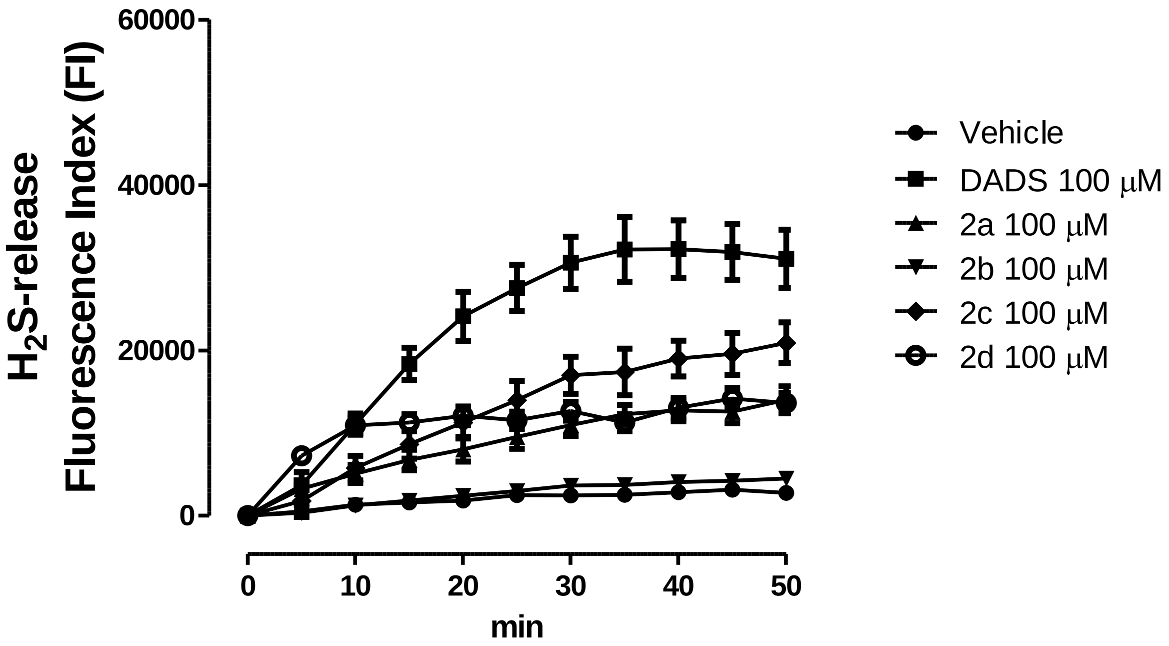

Figure S3. Fluorometric recording of H_2S released by vehicle, DADS 100μM, 2a 100μM, 2b 100μM, 2c 100μM and 2d 100μM for 50 minutes; the increase of H_2S is expressed as fluorescence index (FI).

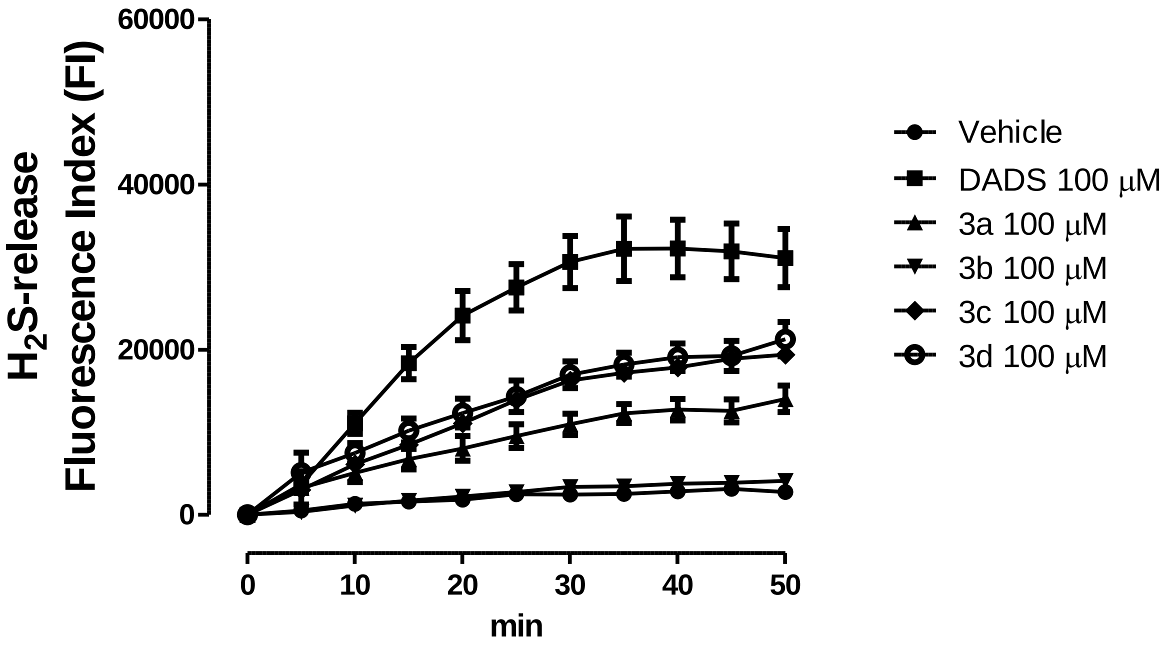

Figure S4. Fluorometric recording of H_2S released by vehicle, DADS 100μM, 3a 100μM, 3b 100μM, 3c 100μM and 3d 100μM for 50 minutes; the increase of H_2S is expressed as fluorescence index (FI).
